# Chloroplast-Localized Protein, OsAL7, with Two Elongation Factor Thermostable Domains Is Essential for Normal Chloroplast Development and Seedling Longevity in *Oryza sativa*

**DOI:** 10.3390/plants14111634

**Published:** 2025-05-27

**Authors:** Jingjing Zhang, Gaokun Chen, Guohua Guan, Yicai Huang, Yunlong Liu, Tingting Xu, Tong Chen, Zemin Zhang

**Affiliations:** 1 State Key Laboratory for Conservation and Utilization of Subtropical Agro-Bioresources, Guangdong Provincial Key Laboratory of Plant Molecular Breeding, South China Agricultural University, Guangzhou 510642, China2022215002@stu.scau.edu.cn (G.C.); 609994023@stu.scau.edu.cn (G.G.); 1362731355@stu.scau.edu.cn (Y.L.);; 2 Guangdong Provincial Key Laboratory of Utilization and Conservation of Food and Medicinal Resources in Northern Region, Shaoguan University, Shaoguan 512005, China

**Keywords:** rice, *albino leaf 7* (*al7*) mutant, albino lethal, elongation factor, chloroplast development

## Abstract

Chloroplast development is crucial for the growth and development of higher plants. In this study, we explored the role of a newly identified factor in this process by using the rice *albino leaf 7* (*al7*) mutant, characterized by an albino phenotype and lethality after the three-leaf stage. Phenotypic analysis indicated that the *al7* mutant exhibited a decreased chlorophyll content and impaired chloroplast development. Using the MutMap+ method, complementation tests, and CRISPR–Cas9 gene editing, we identified that a mutation in the *OsAL7* gene is responsible for the lethal albino phenotype. *OsAL7* encodes a chloroplast-localized protein featuring two elongation factor thermostable (EF-Ts) domains and is expressed ubiquitously across various rice tissues. Deletion of the EF-Ts domains led to defective chloroplast development and albino lethality in seedlings. Moreover, the expression levels of nuclear and plastid genes related to chloroplast development were substantially altered in the *al7* mutant. In conclusion, our findings highlight the key role played by *OsAL7* in the development of chloroplasts and survival of rice seedlings.

## 1. Introduction

In plants, the chloroplasts serve as the main site for photosynthesis, which is essential for the synthesis of carbon required for plant growth, development, and human nutrition [1,2,3,4]. Impaired chloroplast development can lead to diverse abnormal phenotypes, including embryo lethality and pigment deficiencies such as albinism and stunted growth [5,6,7,8,9]. As semi-autonomous organelles, chloroplasts develop under the control of both plastid and nuclear genes [10,11]. In higher plants, chloroplast development is considered to occur in three stages: plastid DNA synthesis and division, plastid transcription and translation machinery establishment, and photosynthetic apparatus activation [12,13,14,15]. In this process, chloroplast genome-encoded genes are transcribed by two RNA polymerases: a nuclear-encoded RNA polymerase (NEP) and a plastid-encoded RNA polymerase (PEP) [7,16]. NEP is transported into the plastids to transcribe various genes, such as *rpoA*, *rpoB*, *rpoC1*, and *rpoC2*, which encode the four core subunits of PEP. Consequently, PEP participates in the transcription and translation of other chloroplast genes [17,18,19]. Disruption in the function of either NEP or PEP affects the transcription of these genes, leading to abnormal chloroplast development and impaired plant growth [7,20,21,22,23].

Ribosomes are crucial for chloroplast development and plant growth because they facilitate the transcription and translation of plastid-encoded proteins [14]. The 70S-type ribosome, similar to those found in prokaryotes, consists of a 50S large subunit and a 30S small subunit [24]. During chloroplast development, most proteins are synthesized outside the chloroplast and then transported into the chloroplast, whereas few proteins are translated directly by chloroplast ribosomes [25,26,27]. Mutations in the genes related to chloroplast ribosomal subunits can hinder chloroplast development, leading to changes in leaf color in rice. For instance, mutations in *ASL1*, *ASL2*, and *WLP1*, which encode the plastid ribosomal small subunit S20, large subunit L21, and large subunit L13, respectively, can impair chloroplast development and confer the plants an albino phenotype [28,29,30,31]. These findings indicate the vital role of plastid ribosomes in chloroplast development.

During the translation process in chloroplast ribosomes, elongation factors, such as EF-Tu and EF-Ts, play critical roles in incorporating aminoacyl-tRNAs (aa-tRNAs) into the ribosomals [32,33]. In rice, the *TSA* gene has been reported to encode the chloroplast elongation factor EF-Tu. Mutations in this gene obstructed the translation of chloroplast genes, indicating that EF-Tu is primarily responsible for chloroplast protein synthesis [33]. In *Chlamydomonas reinhardtii*, the polyprotein of EF-Ts (PET), which is likely post-translationally processed into the chloroplast PSRP-7 and EF-Ts, has been studied for its functional significance [34]. The EF-Ts domain exhibits potential functions in chloroplasts by interacting with the EF-Tu domain [34]. In *Arabidopsis*, EF-Tu and EF-Ts are encoded by the genes *SVR11* and *EMB2726*, respectively, and have been demonstrated to play crucial roles in chloroplast development and embryogenesis [8,35].

The objective of this study is to enrich the regulatory network of chloroplast development. At first, a stable *al7* mutant was characterized by a lethal albino phenotype, decreased chlorophyll content, and defective chloroplast structures. In addition, the *OsAL7* gene was mapped using the MutMap+ approach, and transformation experiments revealed that EF-Ts domains are crucial for the survival of rice seedlings. Furthermore, the expression of genes involved in chloroplast development and chlorophyll biosynthesis was detected. These findings indicate that OsAL7 is vital for the development of the chloroplasts and survival of rice seedlings, which will be useful for enriching the regulatory mechanisms of chloroplast development in rice.

## 2. Results

### 2.1. al7 Mutant Exhibits a Seedling-Lethal Phenotype

**Figure 1:**
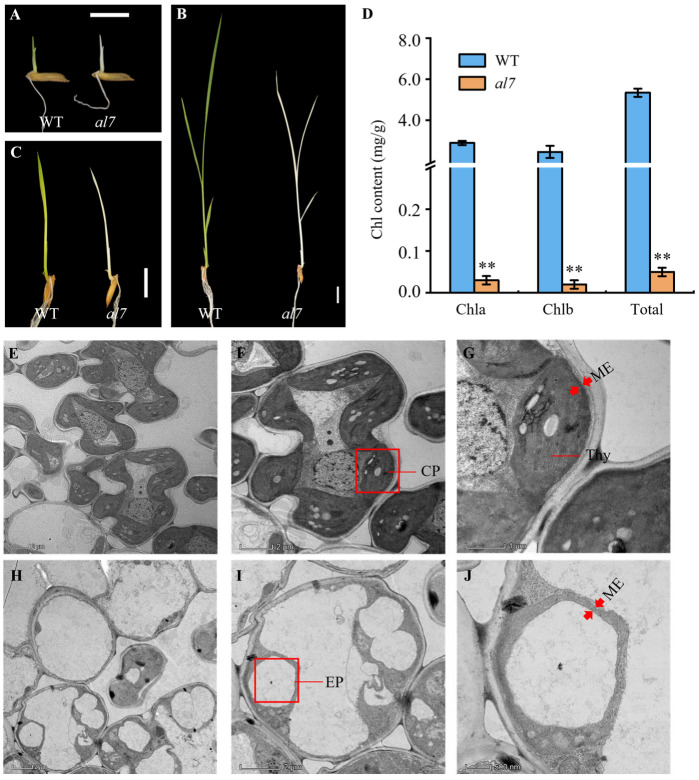
Phenotypic characteristics of WT and *al7* mutant. (**A**–**C**) Phenotypes of WT and *al7* mutant at 3, 7, and 14 days after germination; seedlings were grown in an artificial incubator at 28 °C. Scale bar = 1 cm. (**D**) Chlorophyll (Chl) content in WT and *al7* mutant seedlings at the three-leaf stage. Data are presented as the mean ± SD from three biological replicates. Student’s *t* test was used to determine significant differences; ** indicates a significant difference at the 0.01 level between WT and *al7* mutant. Chl a, chlorophyll a; Chl b, chlorophyll b; and Total, total chlorophyll. (**E**–**J**) Transmission electron microscopy images of third leaves from WT (E, G) and *al7* mutant seedlings (**H**,**J**) at the three-leaf stage. Red arrows represent the membranes envelope of chloroplast or etioplast. Scale bar = 5 μm in E, H; 2 μm in F, I; 1 μm in G, J. cp, chloroplast; thy, thylakoid; ep, etioplast; me, membranes envelope.

In this study, we identified a stable *al7* mutant from an ethyl methanesulfonate (EMS)-mutagenized library of the Xian/Indica cultivar R2B created in our laboratory (Figure S1). Compared with the wild-type (WT) R2B, the *al7* mutant exhibited an albino phenotype starting at three days after germination, and it survived until the three-leaf stage (Figure 1A–C). Moreover, this phenotype resembled that of other albino mutants previously identified in our studies, including *al1*, *al2*, and *al4* [36,37,38]. The *al7* seedlings gradually withered and eventually died. Consistent with their phenotypes, chlorophyll *a*, chlorophyll *b*, and total chlorophyll levels were significantly lower in the *al7* mutant than in the WT rice plants (Figure 1D). These results suggest that the seedling-lethal phenotype of the *al7* mutant is likely due to the impaired chlorophyll accumulation.

### 2.2. Chloroplast Development Is Impaired in the al7 Mutant

In plants, chlorophyll levels are closely linked to chloroplast biogenesis. To explore this further, we used transmission electron microscopy (TEM) for examining and comparing the chloroplast structures of WT and *al7* seedlings at the three-leaf stage. The results of TEM showed that the chloroplasts in the mesophyll cells of the wild type developed normally. Further observation of the chloroplast structure revealed that the stroma was filled with abundant and distinct highly stacked thylakoid lamellae (Figure 1E–G). In contrast, the chloroplasts in most of the mesophyll cells of the *al7* mutant developed abnormally. These chloroplasts severely lacked the thylakoid structure, causing the pigment deposition process to be unable to proceed normally. The internal structure of the chloroplasts gradually degenerated, and finally, only the double-membrane structure was left in the end, degenerating into etioplasts that had lost the ability to perform photosynthesis (Figure 1H–J). These results indicated that the albino phenotype of the *al7* mutant was due to a significant inhibition of thylakoid development, accompanied by a disorder in the pigment accumulation process. This hindered the formation of functional chloroplasts and led to their degeneration into etioplasts.

### 2.3. Molecular Cloning of OsAL7

**Figure 2:**
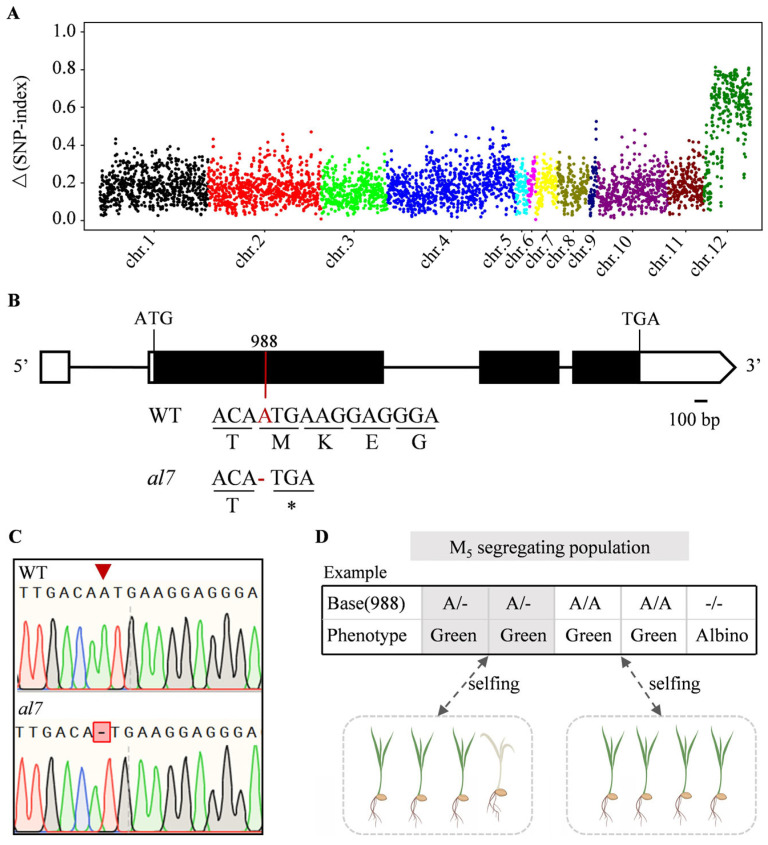
Mapping of *OsAL7*. (**A**) ∆(SNP-index) plot across the entire genome, generated using the MutMap^+^ method. (**B**) Gene structure of *OsAL7* and location of SNP. ATG and TGA indicate start and stop codons, respectively. Black boxes represent exons, white boxes represent untranslated regions (UTRs), and thin black lines represent introns. The location of a single base deletion in the *al7* mutant is highlighted in red, which causes a premature stop codon. The underlined three bases represent codons, and the corresponding amino acids are designated as a single letter, * represents the stop codon. (**C**) Detection of a single base (**A**) deletion in the *al7* mutant through sequencing. (**D**) Co-segregation analysis confirming the association between the genotype and phenotype in a segregating population.

To identify the gene responsible for the albino phenotype in the *al7* mutant, we employed the MutMap+ gene mapping strategy on an M4 segregated population derived from the normal M3 generation plants. Analysis of the segregation ratio of green to albino seedlings, which was 171:41 (χ^2^ 0.05 = 3.62 < 3.84, *p* = 0.057 > 0.05), indicated that the albino phenotype in the *al7* mutant was caused by a single recessive gene. Subsequently, DNA from 40 normal leaf-colored plants and 40 albino plants from the M4 population was isolated and sequenced. After alignment to the reference genome, we identified 4159 SNPs. Analysis of the Δ(SNP-index) revealed their distribution across the 12 chromosomes of rice, with a notable peak on chromosome 12 (Figure 2A). Closer inspection of chromosome 12 revealed a sharp increase in the Δ(SNP-index) within a 4.53 Mb genomic region, suggesting the presence of the gene causing the *al7* phenotype (Figure S2). SNP filtering by using a standard of Δ(SNP-index) close to 0.5 and a mutant bulk SNP index of ≥0.9 highlighted a 1 bp deletion in the open reading frame of *LOC_Os12g35630*, leading to the premature termination of translation (Figure 2B). To confirm this finding, we PCR-amplified and sequenced the candidate gene in both the WT and the *al7* mutant. This analysis revealed a single base (A) deletion at position +988 in the coding region of *LOC_Os12g35630* in the *al7* mutant (Figure 2B,C).

A co-segregation analysis was conducted on the EMS-derived generations to confirm the association between the identified mutation and the albino leaf phenotype. During the seedling stage of the M5 population, all albino plants exhibited a deletion of one adenine (A) residue in their genotype, whereas all WT plants were either homozygous or heterozygous without any deletion. Subsequent observations in the M6 population revealed that leaf color segregation phenotypes at the seedling stage occurred exclusively in progenies derived from M5 heterozygous individuals. On the basis of these findings, we considered gene *OsAL7* for the rice *albino leaf 7* (*al7*) mutant.

**Figure 3:**
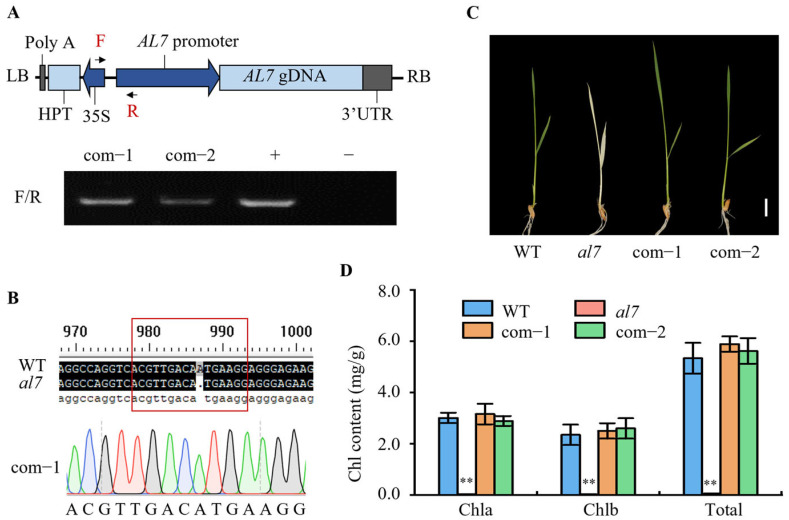
Phenotypic characteristics of complemented *al7* mutants. (**A**) Schematic representation of the complementary vector, and PCR identification of complementary plants. The primers used for PCR are highlighted in red. “+” represents a positive control when using a complementary vector as a template, whereas “−” represents a negative control when using WT DNA as a template. (**B**) Genotype identification of the complementary plant com-1 through sequencing. (**C**) Phenotypes of WT, *al7* mutant, and complementary lines. Scale bar = 1 cm. (**D**) Chlorophyll (Chl) content of WT, *al7* mutant, and complementary lines. Data are presented as the mean ± SD from three biological replicates. Student’s *t* test was used to determine significant differences; ** represents a significant difference at the 0.01 level when compared with WT.

To determine whether *LOC_Os12g35630* is the *OsAL7* gene, we performed a molecular complementation experiment. The 8.39kb full-length genomic sequence of *LOC_Os12g35630*, including its native promoter, was cloned into the pCAMBIA1300 vector. This recombinant plasmid was then transferred into the rice variety Zhonghua11 (Figure 3A). The complementary transgenic lines expressing *OsAL7* were generated by crossing these transgene-positive plants with heterozygous plants (genotype *AL7*/*al7*; Figure 3A and 3B). As expected, the complementary transgenic plants, com-1 and com-2, exhibited normal leaf phenotypes, and their chlorophyll content was comparable to that of WT plants (Figure 3C,D). These results demonstrated that the albino phenotype of plants with the genotype *al7*/*al7* could be rescued by introducing the WT *OsAL7* gene. On the basis of these observations, we confirmed that *LOC_Os12g35630* is the *OsAL7* gene.

### 2.4. OsAL7 Is a Chloroplast-Localized Protein Containing Two EF-Ts Domains

**Figure 4:**
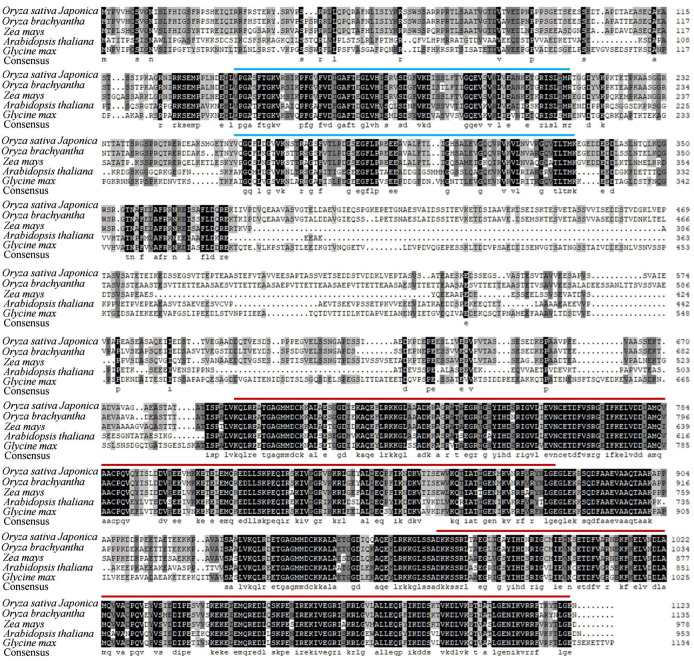
Amino acid sequence alignment of OsAL7 and its closest homologs. Conserved amino acids are highlighted with black and gray backgrounds. Blue lines above the sequences indicate S1 domains, whereas red lines represent EF-Ts domains. GenBank database accession numbers: *Oryza sativa Japonica* (XP_015619919.1), *Oryza brachyantha* (XP_040385682.1), *Zea mays* (NP_001335682.1), *Arabidopsis thaliana* (NP_001031743.1), and *Glycine max* (XP_003534213.1).

**Figure 5:**
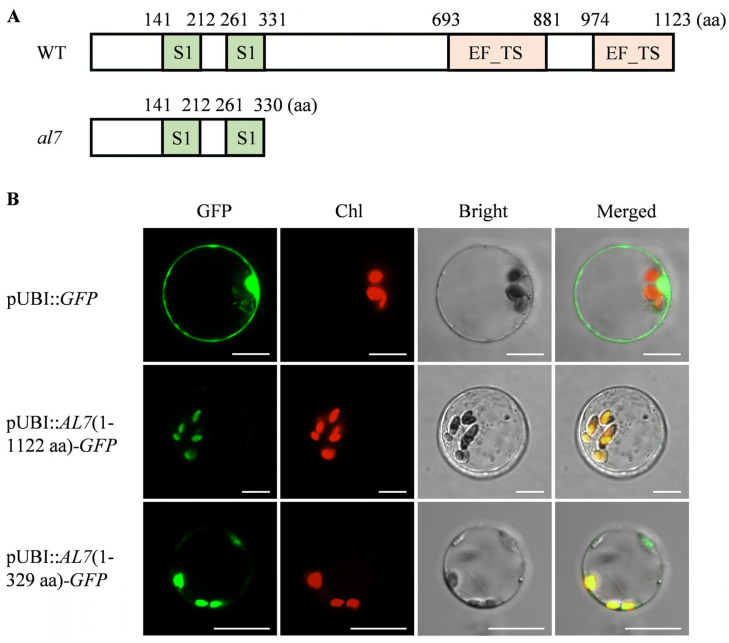
Subcellular localization of full-length and truncated OsAL7 protein. (**A**) Predicted structure of the OsAL7 protein from WT and the *al7* mutant. EF-Ts: elongation factor Ts domain. (**B**) Subcellular localization of the full-length and truncated version of OsAL7. GFP, green fluorescent protein signals; Chl, chloroplast autofluorescence signals; Bright, bright-field image; Merged, merged image of GFP, Chl, and Bright. Scale bar = 10 μm.

Sequence analysis revealed that *OsAL7* comprised three exons and a coding sequence of 3372 bp, which encodes a 1123-amino acid protein. This predicted protein contained two S1 domains and two EF-Ts domains (Figure 4 and Figure S3). A single base deletion at position 988 of an A residue was identified in the first exon of *OsAL7*. This deletion led to the premature termination of translation, resulting in a truncated protein of 330 amino acids. In the *al7* mutant, the truncated protein retained the two S1 domains but lacked the two EF-Ts domains (Figure 5A). Phylogenetic analysis indicated that proteins homologous to OsAL7 are commonly present in both monocotyledons and dicotyledons (Figure S4). Amino acid sequence alignment demonstrated that OsAL7 shares 73%, 64%, and 44% similarity with the homologous proteins of *Zea mays*, *Arabidopsis thaliana*, and *Glycine max*, respectively (Figure 4). The similarity between *OsAL7* and *EMB2726* in *A. thaliana* suggested the involvement of *OsAL7* in chloroplast biogenesis.

UniProt (https://www.uniprot.org/uniprotkb (accessed on 15 February 2023)) predicted that OsAL7 was localized to chloroplasts or plastids. To confirm the subcellular localization of OsAL7, we constructed a vector expressing an OsAL7-GFP fusion protein and introduced it into rice protoplasts. Confocal laser-scanning microscopy analysis of these transformed protoplasts revealed that the green fluorescence from both the full-length and truncated OsAL7-GFP fusion proteins coincided with the autofluorescence of chlorophyll in the chloroplasts (Figure 5B). These observations suggested that both the complete and truncated versions of OsAL7 were localized to the chloroplast, and deletion in the C terminus of OsAL7 did not affect its subcellular localization.

### 2.5. EF-Ts Domains of OsAL7 Are Critical for Its Function

**Figure 6:**
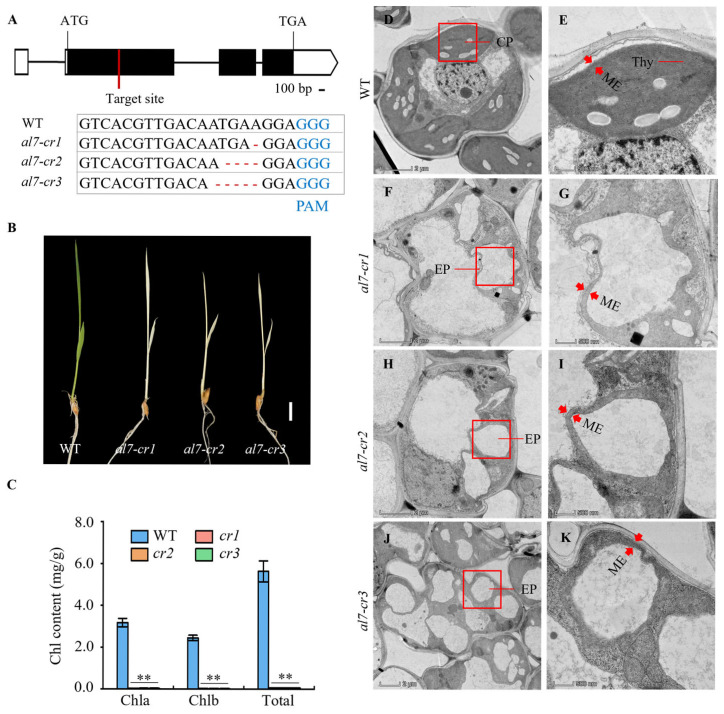
Generation and characterization of rice (*Oryza sativa*) *al7* mutants by using the CRISPR–Cas9 system. (**A**) Sequences around the target in WT and homozygous *al7* mutants. CRISPR–Cas9 editing sites are indicated by red lines. “-” represents a deletion; PAM: Protospacer adjacent motifs. (**B**) Phenotypes of WT, *al7*-1, *al7*-2, and *al7*-3 seedlings at the two-leaf stage. Scale bar = 1 cm. (**C**) Chlorophyll content of WT, *al7-cr1*, *al7-cr2*, and *al7-cr3* seedlings at the three-leaf stage. Asterisks indicate a significant difference when compared with the WT, as determined using Student’s *t* test (** *p* < 0.01). (**D**–**K**) Transmission electron microscopy images of cells from WT, *al7-cr1*, *al7-cr2*, and *al7-cr3* seedlings at the third-leaf stage. Red arrows represent the membranes envelope of chloroplast or etioplast. Scale bar = 2 μm in (**D**,**F**,**H**,**J**); 0.5 μm in (**E**,**G**,**I**,**K**). cp, chloroplast; ep, etioplast; thy, thylakoid; me, membranes envelope.

The amino acid sequences of EF-Ts domains are highly conserved across plants, indicating their critical role in the functionality of OsAL7 (Figure 4). OsAL7 is homologous to the PETs, which contain two S1 domains, named PSRP-7, and two EF-Ts domains, at the carboxyl end [34]. The PET precursor protein is likely posttranslationally processed into two polypeptides: the 65-kD PSRP-7 and the 55-kD EF-Ts [34]. To investigate the role of the EF-Ts domains in chloroplast biogenesis, three independent homozygous mutants (named *al7*-1, *al7*-2, and *al7*-3) lacking the EF-Ts domains were created using the CRISPR/Cas9 technique. These mutants were characterized by three mutations: a single base (A) deletion at position 992, a four-base (TGAA) deletion at position 989, and a five-base (ATGAA) deletion at position 988, leading to frameshift mutations that resulted in the premature termination of the encoded protein, retaining only the two S1 domains (Figure 6A and Figure S5). These mutants displayed an albino phenotype and eventually died (Figure 6B). Consistent with their phenotypes, chlorophyll *a*, chlorophyll *b*, and total chlorophyll levels were significantly decreased in the *OsAL7*-Cas9 lines (*al7*-1, *al7*-2, and *al7*-3) compared with the WT (Figure 6C). However, heterozygous mutants exhibited normal green leaves and growth, with leaf color phenotype segregation observed in their progeny (Figure S6).

To investigate the effect of *al7* mutation on chloroplast biogenesis, we examined the chloroplast ultrastructure at the three-leaf stage by using TEM. The observation results were revealed that the chloroplasts in the leaves of the wild type developed well, had a normal morphology, and the thylakoid structure was clear (Figure 6D–E). The chloroplasts of the *OsAL7*-Cas9 mutants (*al7*-1, *al7*-2, and *al7*-3) were shown to exhibit obvious developmental abnormalities, which were characterized by the degeneration of chloroplasts into etioplasts, the lack of thylakoid structures, with only the double-membrane structure being retained, and, thus, no pigment accumulation occurred. This ultimately led to the photosynthetic function of the plants being lost and the albino phenotype being presented (Figure 6F-K). These results highlighted the importance of the C-terminal structure of the OsAL7 protein, including the EF-Ts domain, in the development of chloroplasts and the survival process of rice seedlings.

### 2.6. Expression Pattern of OsAL7

**Figure 7:**
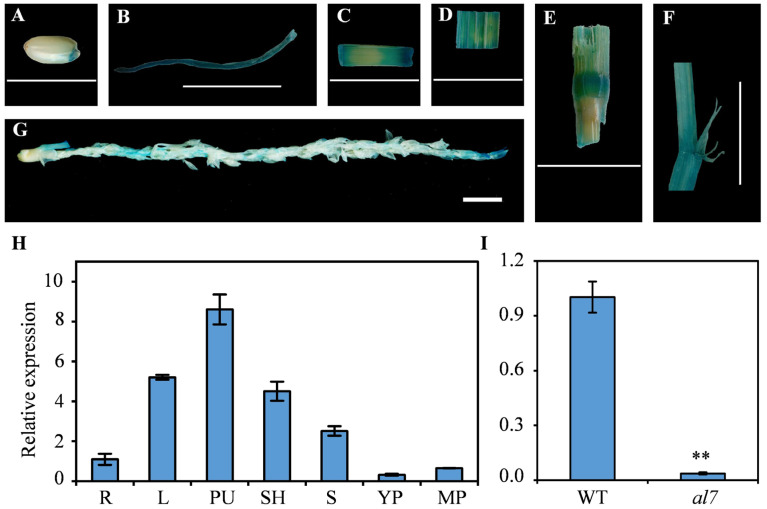
Expression pattern of *OsAL7*. (**A**–**G**) GUS staining of the embryo at two days after germination (**A**), roots (**B**), stems (**C**), leaves (**D**), nodes (**E**), leaf ligules and auricular auricles (**F**), and young panicles (**G**) from *pAL7^R2B^::GUS* transgenic seedlings. Scale bar = 1 cm. (**H**) *OsAL7* transcription level in various organs. R, roots; L, leaves; PU, pulvinus; SH, leaf sheaths; S, stems; YP, young panicles; and MP, mature panicles. (**I**) *OsAL7* transcription level in the WT and *al7* mutant seedlings. *Ubiquitin* was used as the reference gene. Student’s *t* test was used to determine significant differences; ** represents a significant difference between the WT and *al7* mutant at the 0.01 level.

To examine the expression patterns of *OsAL7*, transgenic rice plants harboring the *pOsAL7::GUS* reporter construct were developed using the *Agrobacterium*-mediated method. GUS expression was detected across various tissues via staining, confirming the ubiquitous presence of *OsAL7*. GUS was expressed in young embryos, roots, stems, leaves, nodes, leaf sheaths, and young panicles, suggesting that *OsAL7* functions throughout the plant’s life cycle (Figure 7A–G). Furthermore, GUS staining results were consistent with the expression profiles identified by qRT-PCR, showing *OsAL7* activity in diverse tissues, including roots, stems, leaves, leaf sheaths, pulvinus, young panicles, and mature panicles (Figure 7H). Among them, pulvinus exhibited the highest expression level of *OsAL7*, followed by leaves and leaf sheaths (Figure 7H). These results demonstrated that *OsAL7* is constitutively expressed in various tissues and mainly functions in green tissues. Moreover, the expression level of *OsAL7* was significantly lower in the *al7* mutant than in the WT (Figure 7I). These findings highlight the pivotal role of *OsAL7* in chloroplast development and overall plant growth.

### 2.7. Mutation in OsAL7 Affects the Transcription of Chloroplast-Associated Genes

**Figure 8:**
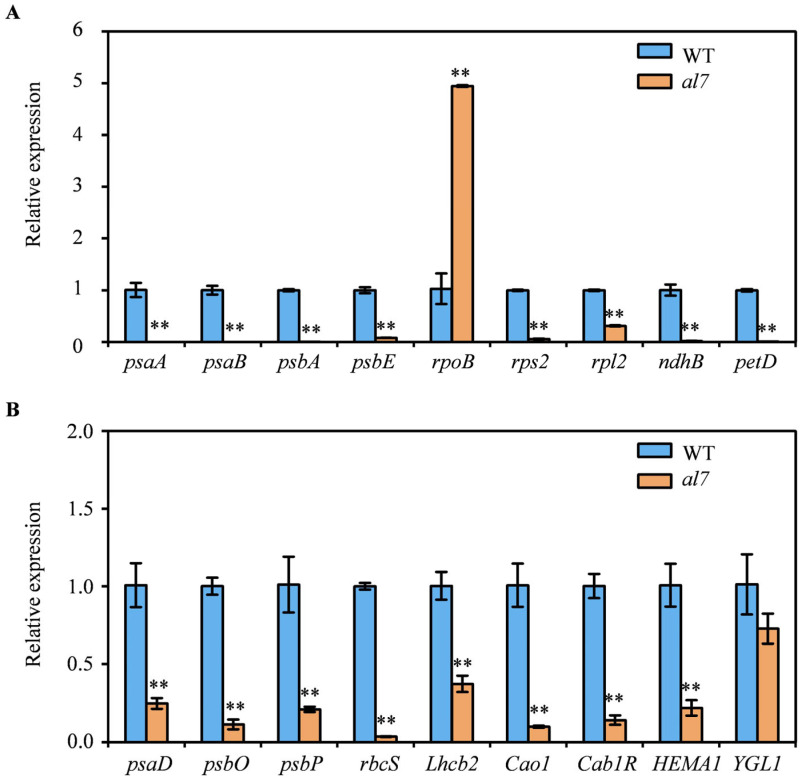
Relative expression levels of genes related to chloroplast development in WT and *al7* mutant. (**A**) Relative expression levels of plastid-encoded genes associated with chloroplast development in the WT and *al7* mutant. ** represents a significant difference between the WT and *al7* mutant at the 0.01 level. (**B**) Relative expression levels of nucleus-encoded genes associated with photosynthesis and chlorophyll biosynthesis in the WT and *al7* mutant. ** represents a significant difference between the WT and *al7* mutant at the 0.01 level.

The development of chloroplasts involves an intricate coordination between the nuclear and chloroplast genes. Given that the *OsAL7* mutation caused abnormal chloroplast structures, we examined the expression of both nuclear-encoded and plastid-encoded genes associated with chloroplast development in the WT and *al7* mutant by using qRT-PCR. The transcript levels of PEP-encoded photosynthesis genes, such as *psaA*, *psaB*, *psbA*, *psbE*, and *petD*, were markedly decreased in the *al7* mutant, almost approaching zero, suggesting a decrease in PEP activity (Figure 8A). By contrast, the expression of the NEP-dependent gene *rpoB*, which encodes the β-subunit of PEP, was significantly increased in the *al7* mutant (Figure 8A). In addition, the expression levels of the plastid genes *rpl2* and *rps2*, which are involved in ribosome synthesis, were significantly decreased in the *al7* mutant (Figure 8A).

Transcript levels of the nuclear genes involved in photosynthesis, such as *psaD*, *psbO*, *psbP*, *rbcS*, and *Lhcb2*, were significantly lower in the *al7* mutant than in the WT (Figure 8B). Similarly, the expression of nuclear-encoded chlorophyll biosynthesis genes, including *Cao1*, *Cab1R*, and *HEMA1*, was remarkably lower in the *al7* mutant than in the WT. However, the transcript levels of *YGL* did not significantly differ between the *al7* mutant and WT (Figure 8B). These findings indicated that the mutation in *OsAL7* disrupted the expression of genes associated with chloroplast development and chlorophyll biosynthesis, contributing to the albino phenotype observed in the *al7* mutant.

## 3. Discussion

Numerous albino mutants have been identified in rice, including *al1*, *al2*, *las1*, *asl1*, and *asl4* [14,28,36,37,39,40]. Although these mutants are regulated by different genes, they share common traits, such as decreased chlorophyll content, impaired chloroplast development, and early seedling lethality. In the present study, we isolated and characterized the *al7* mutant, which exhibited a lethal albino phenotype. Further investigations revealed that the *al7* mutant had substantial defects in chlorophyll accumulation and chloroplast ultrastructure (Figure 1). Gene mapping, cloning, and complementation studies led to the identification of a new gene, named *OsAL7*. A single base (A) deletion in this gene was found to cause the lethal albino phenotype observed in the *al7* mutant (Figure 2 and Figure 3).

Mutation in *OsAL7* led to abnormal chloroplast structures, suggesting impaired chloroplast development in the *al7* mutant (Figure 1G,H). Chloroplast development is regulated through the coordination between the nuclear and plastid genes. Similar abnormalities in gene expression associated with chloroplast development have been observed in other leaf color mutants, including *wsl4*, *pgl12*, and *asl4* [3,7,19,37,41,42,43,44]. In the case of *OsAL7* mutation, we noted a disruption in the expression of both nuclear and plastid genes that are crucial for chloroplast development (Figure 8).

While OsAL7 is ubiquitously expressed in various tissues, the phenotypic defects are predominantly observed in early-stage green tissues. This tissue specificity may reflect the heightened demand for chloroplast activity and translational capacity in developing leaves compared to other organs.

The chloroplast is a semi-autonomous organelle equipped with its own transcription and translation systems. Chloroplast genes are transcribed coordinately by two types of RNA polymerases: PEP and NEP [7,45]. When the PEP complex is dysfunctional, the expression of PEP-dependent genes decreases. This complex comprises four core subunits, encoded by the plastid genes *rpoA*, *rpoB*, *rpoC1*, and *rpoC2*, respectively [46]. For instance, the knockout of *OsPPR16* suppresses *RpoB* accumulation and reduces the transcription of PEP-dependent genes [7]. The decreased transcript levels of PEP-dependent photosynthesis genes (*psaA*, *psaB*, *psbA*, *psbE*, and *petD*) in the *al7* mutant suggest that PEP activity of the *al7* mutant is compromised, potentially causing the increased expression of the NEP-dependent gene *rpoB* (Figure 8A). Similar expression patterns in plastid genes have been observed in mutants with defective PEP such as *wsp2*, *wsl4*, and *cde4* [3,19,47]. These findings indicated that *OsAL7* is crucial for the formation of the chloroplast PEP transcription machinery, and disruptions in the chloroplast transcriptional/translational apparatus occurred in the *al7* mutant. Overall, *OsAL7* plays an essential role in the development of chloroplasts and survival of rice seedlings.

The chloroplast translation system in plants shares similarities with that of prokaryotes. The elongation factors EF-Tu, EF-G, and EF-Ts are the key to the peptide chain elongation [32]. The EF-Ts domain, extensively studied in prokaryotes, serves as a guanine nucleotide exchange factor for the EF-Tu domain during translation [48,49,50,51,52,53]. In rice, the gene *TSA*, encoding the chloroplast elongation factor EF-Tu, is primarily involved in the synthesis of chloroplast proteins [33]. Our study revealed that the albino phenotype observed in the *al7* mutant is caused by the mutation in *OsAL7*, which encodes a 1123-amino acid protein predicted to contain two S1 domains and two EF-Ts domains (Figure 2 and Figure S3).

OsAL7, a homolog of PETs, is likely post-translationally processed into two components: PSRP-7, containing two S1 domains, and the EF-Ts polypeptide [34]. PSRP-7 constitutes the 30S subunit and can bind to chloroplast mRNAs in vitro [34,54]. Additionally, EF-Ts has been shown to bind EF-Tu in vitro, suggesting its functional role within the chloroplast [34]. However, experimental validation for the processing of OsAL7 into these two components is lacking yet. The conservation of the PET polyprotein across various species, including green algae (*C. reinhardtii*), dicots (*A. thaliana*), and monocots (*Oryza sativa*), implies a collaborative function of PSRP-7 and EF-Ts in the translation process in chloroplasts [34]. As anticipated, the amino acid sequences of EF-Ts were found to be highly conserved among *O. sativa*, *Z. mays*, *A. thaliana*, and *G. max* (Figure 4). Given that *OsAL7* encodes the only putative EF-Ts targeted to the chloroplast in rice (Figure 5), it is highly probable that *OsAL7* functions as a guanine nucleotide exchange factor during translation in plastids.

Despite homology to known elongation factors, our study lacks direct biochemical evidence confirming OsAL7 interaction with EF-Tu or its function in nucleotide exchange. In vitro binding or GTP/GDP exchange assays are necessary to validate this mechanistic role and represent an important avenue for future research. This limitation should be acknowledged when interpreting the proposed function of OsAL7.

Finally, while *OsAL7* is a novel gene, its role closely resembles that of *EMB2726* in *Arabidopsis* and *TSA* in rice [8,33]. The phenotypic and molecular parallels with known albino mutants reinforce a conserved requirement for plastid translational machinery in early chloroplast biogenesis. The single base deletion in *al7* results in a truncated protein lacking EF-Ts domains, leading to chloroplast defects and lethality. CRISPR-induced mutants with similar deletions confirmed these findings. Together, these results highlight OsAL7 as the EF-Ts domain-containing protein targeted to chloroplasts in rice and underscore its essential role in seedling survival and chloroplast development.

## 4. Materials and Methods

### 4.1. Plant Materials and Growth Conditions

The rice *al7* mutant was obtained from the mutant population generated by ethyl methyl sulfonate (EMS) treatment with Xian/Indica cultivar ‘R2B’ background. The segregated population from *AL7*/*al7* heterozygous self-crossed progeny was used to identify the mutant gene. All rice seedlings used in this study were grown in a growth chamber under a 12 h light/12 h darkness cycle at 28 °C.

### 4.2. Chlorophyll Content Measurement

The chlorophyll contents were measured following the method described by Zhou [55]. Briefly, fresh leaves (0.1 g) were collected, cut into small pieces, and incubated in 2 mL of extraction buffer (ethanol/acetone/H_2_O = 4.5:4.5:1, volume ratio) for 48 h at 4 °C. The absorbance of the supernatants was detected at 645 nm and 663 nm using a spectrophotometer (Bio-Rad SmartSpec^TM^ Plus, Irvine, CA, USA).

### 4.3. Transmission Electron Microscopy (TEM)

At the three-leaf stage, leaves from WT and *al7* mutant plants were cut into 0.5 cm slices at the same parts. The tissues were fixed in 2.5% glutaraldehyde at 4 °C for 4 h, rinsed, and incubated overnight in 1% OsO_4_ at 4 °C. After fixation, the samples were subsequently dehydrated in an ethanol series, further infiltrated in a gradient epoxy resin series, and finally embedded in resin. Thin sections were observed using TEM (Talos, Hillsboro, OR, USA).

### 4.4. MutMap+ Method for Mapping the OsAL7 Gene

The MutMap+ method was used to map the *OsAL7* gene according to Fekih [56]. Briefly, a mutant pool of DNA (Pool M) and a WT pool of DNA (Pool W) from 40 white and 40 green seedlings of a segregated population were prepared and subjected to whole-genome sequencing. High-quality clean sequence reads of Pool M and Pool W were aligned to the reference genome R2B, and SNP indexes were calculated. ∆(SNP-index) was obtained by aligning SNPs between Pool M and Pool W. Regions with the standard of ∆(SNP-index) close to 0.5 and a mutant bulk SNP-index ≥ 0.9 were considered for candidate regions of the *al7* mutant phenotype. Finally, the mutation was further verified with cosegregation analysis by amplifying the region containing mutations in green and albino leaf plants with different EMS generations.

### 4.5. Complementation Test

For the complementation test, an 8392 bp genomic fragment, including a 2095 bp upstream sequence, the *OsAL7* genomic sequence, and an 871 bp downstream sequence, was amplified from R2B and cloned into binary vector pCAMBIA1300 to generate plasmid pAL7. This vector was then transformed into a ZH11 background because the callus of the *al7* mutant (an indica variety) was difficult to obtain. Then, positive transgenic plants were crossed with an *Al5/al5* heterozygote, generating complementation plants. All primers used for vector construction and detection are listed in Table S1.

### 4.6. CRISPR/Cas9 Knockout of OsAL7

The CRISPR/Cas9 system was adopted to generate *al7* mutants as previously described [57]. The gRNA target site was designed by the CRISPR-GE website online, and this expression cassette is driven by the *Zea mays* U3 promotor. Then, the gRNA expression cassette was cloned into binary vector pYLCRISPR/Cas9Pubi-H, generating a recombinant plasmid. The CRISPR vectors were introduced into a 9311 background by *Agrobacterium*-mediated transformation. The genotype of *OsAL7*-Cas9 plants was analyzed using direct sequencing of PCR amplification products. All primers used for vector construction and detection are listed in Table S1.

### 4.7. Sequence Alignment Analyses

The predicted full-length OsAL7 protein sequence with 1123 amino acids was obtained from SMART. Homologous sequences of OsAL7 in other plants were identified using the NCBI’s BLASTP tool. Multiple sequence alignments were conducted by DNAMAN software. A neighbor-joining phylogenetic tree was generated based on 1000 bootstrap replicates using MEGA software(Version 11.0.13).

### 4.8. RNA Isolation and qRT-PCR Analysis

Total rice RNA was extracted with an RNA Extraction Kit (TRIgol reagent, Beijing Dingguo, Beijing, China) according to the manufacturer’s instructions. First-strand cDNA was synthesized from 2 μg total RNA using One-Step gDNA Removal and cDNA Synthesis SuperMix (TransGen, Beijing, China). The qRT-PCR was performed with the Applied Biosystems™ SYBR™ Green PCR Mix using a Bio-Rad CFX96 system (Fremont, CA, USA). The rice ubiquitin gene was used as normalization control, and the relative expression levels of target genes were calculated by the 2^−ΔΔCT^ method. All qRT-PCR primers are listed in Table S2.

### 4.9. Subcellular Localization

The full-length and truncated cDNA without the termination codon of *OsAL7* were amplified from WT and *al7* mutant plants, respectively. These fragments were cloned into the C-terminus of GFP in the pRTV vector and transformed into rice protoplasts as previously described [58]. Thereafter, protoplast fluorescent signals were observed using a confocal microscope (Nikon Ai2, Otawara City, Tochigi Prefecture, Japan).

### 4.10. β-Glucuronidase (GUS) Histochemical Staining

Tissues from homozygous *pAL7::GUS* transgenic seedlings were incubated for more than 2 h at 37 °C in a GUS staining solution (Coolaber, Beijing, China). The stained tissues were cleared with 70% ethanol at 80 °C and photographed.

## 5. Conclusions

In this study, we identified a rice mutant, albino leaf 7 (*al7*), characterized by a lethal albino phenotype, decreased chlorophyll content, and defective chloroplast structures. Using the MutMap+ approach, we determined that a single base deletion in the gene *OsAL7* leads to the premature termination of translation of the target protein, which results in the *al7* mutant phenotype. *OsAL7* encodes a chloroplast-localized protein featuring two EF-Ts domains and is ubiquitously expressed in various rice tissues. These EF-Ts domains are crucial for the survival of rice seedlings. In *al7* mutants, the expression of genes involved in chloroplast development and chlorophyll biosynthesis is substantially disrupted. This study underscores the importance of *OsAL7* in normal chloroplast development, providing insights for a detailed investigation into the regulatory mechanisms underlying chloroplast development.

## Data Availability

Original raw data supporting the conclusions of this article will be made available by the authors, without undue reservation.

## Supplementary Materials

The following supporting information can be downloaded at: https://www.mdpi.com/article/10.3390/plants14111634/s1, Figure S1: Phenotypic characteristics of the *al7* mutant; Figure S2: △(SNP-index) plot of chromosome 12 in rice; Figure S3: Gene structure of *OsAL7* and protein structure of *OsAL7*; Figure S4: Phylogenetic tree of OsAL5 and its closest homologues from other species; Figure S5: Amino acid sequences alignment between WT and *OsAL7*-related mutants; Figure S6: Phenotype of heterozygous progeny by CRISPR/Cas9 method. Table S1: Primers for vector construction and genotype detection in this study; Table S2: Primers for qRT-PCR in this studyCaption.
