# Supplementary material for: Chloroplast-Localized Protein, OsAL7, with Two Elongation Factor Thermostable Domains Is Essential for Normal Chloroplast Development and Seedling Longevity in Oryza sativa
Source: Plants (Basel). 2025 May 27;14(11):1634. doi: 10.3390/plants14111634 (PMC12156993; doi:10.3390/plants14111634)
Supplement: Supplementary file 1 [file plants-14-01634-s001.zip › plants-3560538-supplementary.pdf]

SUPPORTING INFORMATION

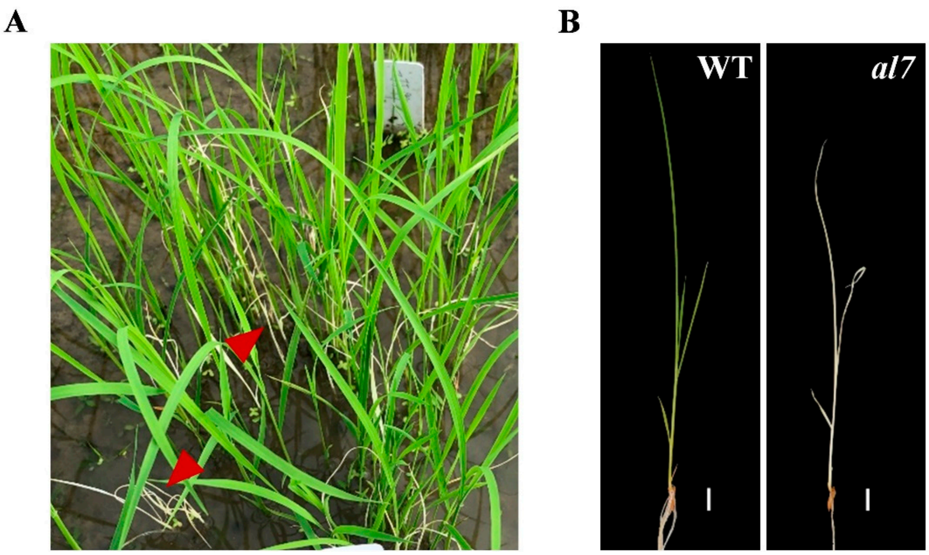

Figure S1. Phenotypic characteristics of the *al7* mutant.

(A) Phenotype of heterozygous progeny at the seeding stage under natural filed conditions, *al7* homozygous plants are indicated using red arrows.

(B) Phenotype of WT (left panel) and *al7* mutant (right panel). Bars=1 cm.

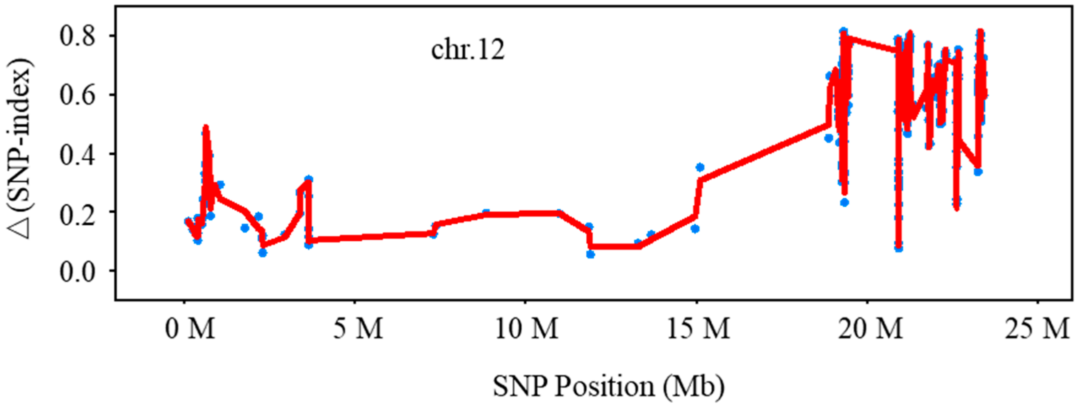

Figure S2. △(SNP-index) plot of chromosome 12 in rice.

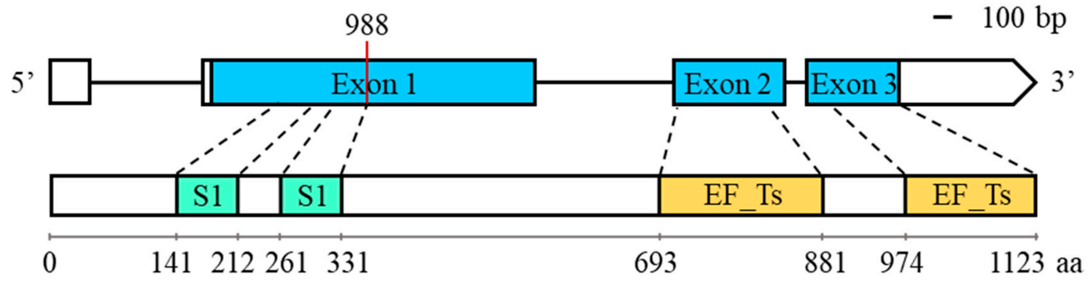

Figure S3. Gene structure of *OsAL7* and protein structure of OsAL7.

The deletion of *al7* mutant at position 988 bp from ATG start codon, resulting in premature termination at amino acid sequences.

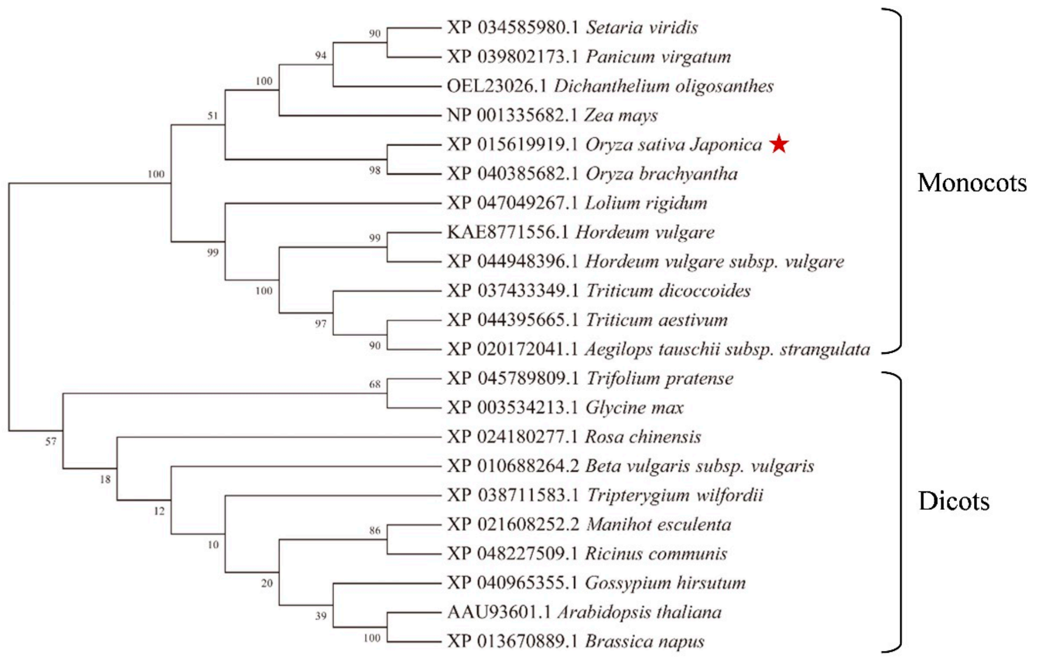

Figure S4. Phylogenetic tree of OsAL5 and its closest homologues from other species.

The phylogenetic tree was constructed by the MEGA software, and numbers near branches represent bootstrap values.

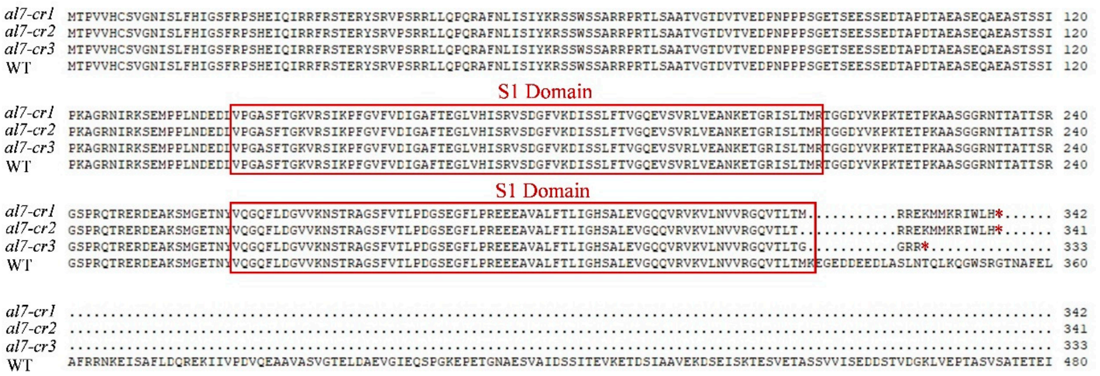

Figure S5. Amino acid sequences alignment between WT and *OsAL7*-related mutants.

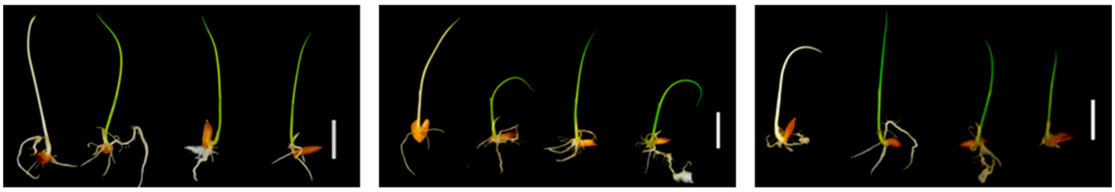

al7-cr1 *al7-cr2* *al7-cr3*

Figure S6. Phenotype of heterozygous progeny by CRISPR/Cas9 method

Phenotypes were observed at the four days after germination from heterozygous, bars=1 cm.

Table S1. Primers for vector construction and genotype detection in this study

Application	Primer	Sequence（5'-3'）
Complementation vector construction	cp-AL7-F1	cagctatgaccatgattacgaattcCCTTATTTCTCTGCACCCGCATC
	cp-AL7-R1	ACTATCAGTCTCCTTGACCTCCG
	cp-AL7-F2	CGGAGGTCAAGGAGACTGATAGT
	cp-AL7-R2	ccaagcttgcatgcctgcag AAGCTTTGAAGAGTGAGCCCTAC
Subcellular localization vector construction	Sub-AL7-F	ccccccgggATGACCCCGGTGGTTCATTGC
	Sub-AL7-R1	ccaagcttGTTCTCACCAAGGGTGTACCG
	Sub-AL7-R2	ccaagcttCTGGCCTCGTACTACGTTC
GUS vector	Pro-AL7-F	aagcttCAGACAAAAGGAATGAAAGCG
	Pro-AL7-R	ggatccTTTCTGTACCCTGCACGAG
Mutmap+ mutant SNP detection	AL7-526-F	GAATACCACTGCAACCACGTC
	AL7-526-R	CTTGCCTGGACTTTGCTCA
Mutmap+ mutant SNP detection	Case9-F	CTGACGCTAACCTCGACAAG
Genotype detection	Case9-R	CCGATCTAGTAACATAGATGACACC
	Com-AL7-F	CGCCAGGGTTTTCCCAGTCACGAC
	Com-AL7-R	GCGGGCATATATCTACCCTC
	CR-AL7-F	GAATACCACTGCAACCACGTC
	CR-AL7-R	CTTGCCTGGACTTTGCTCA

Table S2. Primers for qRT-PCR in this study

Gene	F Primer (5'-3')	R Primer (5'-3')
AL7	AAGAAGTAGCAGCCTCAAGTG	CCCCTGATTCTGCAAGAGCC
rpl2	TGTTGAGTAACGGCAGCAAG	AACCCGTCCTCCTCTCTTTG
psaA	TTAGAAATCCGCCAATCCA	TGCTAGGCTCTACAACCATT
psbE	CCGCCAAGCCGCCTCCCATT	AGCTCGACGACGATCCATCC
psbA	ACCCTCATTAGCAGATTCGT	GATTGTATTCCAGGCAGAGC
psaB	GAGCAATATCGGTCAGCCACA	ACCACTCAAGGAGCGGGAAC
rps2	GAGATGATAGAAGCGGGAGTT	TAACATAATGACAACGAGCC
psaD	CCGCTCCAAGTACAAGATCA	AAGAGCAGCCTGACAGATGA
psbP	AAGACAGATTCCGAGGGTGG	TGATTCGCTAGGGATTAAAGAG
psbO	GCTCTACCGGCTACGACAAC	TGACATCCTTGGGCACCTT
petD	GATCCGTTTGCAACTCCTCT	CCATTAAGAGAACGCCCAAT
ndhB	CCCCTTTTCATCAATGGACT	TGAAGCAGCAACTTTCGAAG
rbcS	TGAGGGCATCAAGAAGTT	CGATGATACGGACAAAGG
lhcb2	CCCCATCGAGAACCTCTTC	CGGTGCGTGGCTACTACAA
Cao1	CAGAATCCAATGCCCTTAC	AGTATCCTTGAAGCCCAGA
HEMA1	CGCTATTTCTGATGCTATGGGT	TCTTGGGTGATGATTGTTTGG
Cab1R	AGATGGGTTTAGTGCGACGAG	TTTGGGATCGAGGGAGTATTT
YGL1	TCTTGGTGCGAGCTACATTG	GCTTGCCTGAACTGAAAAGG
rpoB	TGGTACATATCCCTTATCTCAA	CTCCAGGACCCAAACAACTC
UBQ	TGGTCAGTAATCAGCCAGTTTGG	GCACCACAAATACTTGACGAACAG
